# The effect of successive summer drought periods on bacterial diversity along a plant species richness gradient

**DOI:** 10.1093/femsec/fiae096

**Published:** 2024-07-02

**Authors:** Yuri Pinheiro Alves de Souza, Roberto Siani, Cynthia Albracht, Yuanyuan Huang, Nico Eisenhauer, Anja Vogel, Cameron Wagg, Michael Schloter, Stefanie Schulz

**Affiliations:** Research Unit Comparative Microbiome Analysis, Helmholtz Zentrum München, D-85764 Neuherberg, Germany; TUM School of Life Science, Chair of Environmental Microbiology, Technische Universität München, 85354 Freising, Germany; Research Unit Comparative Microbiome Analysis, Helmholtz Zentrum München, D-85764 Neuherberg, Germany; TUM School of Life Science, Chair of Environmental Microbiology, Technische Universität München, 85354 Freising, Germany; Swammerdam Institute of Life Sciences at University of Amsterdam, 1098 XH Amsterdam, the Netherlands; Department Soil Ecology, Helmholtz Centre for Environmental Research – UFZ, 06120 Halle (Saale), Germany; German Centre for Integrative Biodiversity Research (iDiv) Halle-Jena-Leipzig, 04103 Leipzig, Germany; Institute of Biology, Leipzig University, 04103 Leipzig, Germany; German Centre for Integrative Biodiversity Research (iDiv) Halle-Jena-Leipzig, 04103 Leipzig, Germany; Institute of Biology, Leipzig University, 04103 Leipzig, Germany; German Centre for Integrative Biodiversity Research (iDiv) Halle-Jena-Leipzig, 04103 Leipzig, Germany; Institute of Biology, Leipzig University, 04103 Leipzig, Germany; Department of Geography, Remote Sensing Laboratories, University of Zürich, Winterthurerstrasse 190, CH-8057 Zürich, Switzerland; Fredericton Research and Development Centre, Agriculture and Agri-Food Canada, 95 Innovation Road, Post Office Box 20280, Fredericton E3B 4Z7 NB, Canada; Research Unit Comparative Microbiome Analysis, Helmholtz Zentrum München, D-85764 Neuherberg, Germany; TUM School of Life Science, Chair of Environmental Microbiology, Technische Universität München, 85354 Freising, Germany; Research Unit Comparative Microbiome Analysis, Helmholtz Zentrum München, D-85764 Neuherberg, Germany

**Keywords:** Acidobacteriota, drought recovery, plant diversity, soil microbiome

## Abstract

Drought is a major stressor to soil microbial communities, and the intensification of climate change is predicted to increase hydric stress worldwide in the coming decades. As a possible mitigating factor for the consequences of prolonged drought periods, above and belowground biodiversity can increase ecosystem resistance and resilience by improving metabolic redundancy and complementarity as biodiversity increases. Here, we investigated the interaction effect between plant richness and successive, simulated summer drought on soil microbial communities during a period of 9 years.To do that, we made use of a well-established biodiversity experiment (The Jena Experiment) to investigate the response of microbial richness and community composition to successive drought periods alongside a plant richness gradient, which covers 1-, 2-, 4-, 8-, 16-, and 60-species plant communities. Plots were covered from natural precipitation by installing rain shelters 6 weeks every summer. Bulk soil samples were collected 1 year after the last summer drought was simulated. Our data indicate that bacterial richness increased after successive exposure to drought, with the increase being stable along the plant richness gradient. We identified a significant effect of plant species richness on the soil microbial community composition and determined the taxa significantly impacted by drought at each plant richness level. Our data successfully demonstrates that summer drought might have a legacy effect on soil bacterial communities.

## Teaser

Here, we demonstrated that soil bacterial community increases after long term successive drought periods in a plant richness gradient.

## Introduction

As a result of the intensification of human activities over the past two centuries, Earth´s biosphere is facing unprecedented alterations. Climate change, resulting from increasing industrialization and air pollution, has been considered as the major driver for the change in rainfall pattern across the globe (Fowler and Hennessy 1995, Nemecek et al. 2012, Sohoulande Djebou and Singh 2016). As a consequence of the disturbances on the rain regime, many regions are now experiencing long periods of drought, suffering from insufficient precipitation or impairment of the water distribution of rivers, lakes, and other water bodies (Eriyagama et al. 2009). This hydric stress directly affects agricultural production, threatening the food supply chain (Osborne et al. 2013, Ostad-Ali-Askari et al. 2020, Wang et al. 2020), as well as the equilibrium of natural environments (Huntington 2006, Geng et al. 2015).

The consequences of changes in water availability can be observed on all tropic levels. Whereas data on the responses of plants and animals towards drought stress are available, still we are lacking a clear picture how microbes in soil are influenced by a lack of available water, although microbes can be considered as the architects of soil quality (Vejan et al. 2016, Docherty and Gutknecht 2019). To predict how microbial communities change in response to drought stress is also difficult because of the complexity of the soil microbiome and the differences in ecophysiology of the single microbiota.

Bacterial communities respond very quickly to environmental stress caused by both changes in physicochemical and biological factors, such as increasing temperature, lack of water, or a secondary response to a biotic factor responding to a physicochemical factor (Jansson and Hofmockel 2020). During water stress, bacterial cells increase intracellular solute production to achieve an osmotic equilibrium with the environment, which requires increased energy demands for the cell. The same happens during rewetting, when cells release excess solutes into the environment to achieve osmotic balance (Csonka 1989). The lack of water also reduces bacterial motility and nutrient uptake (Schimel et al. 2007), since the environment becomes less homogenous as water concentrations diminish. Currently, literature on the effects of drought on bacterial communities indicates that drought events are followed on the one hand by an increase in Gram-positive bacteria (such as Actinobacteria), which can utilize recalcitrant carbon sources and are highly present in arid, nutrient-poor soils (Connon et al. 2007). Many of these bacterial taxa are capable to generate stress-resistant structures, like spores (Zeigler 2014). Gram-negative bacteria, on the other hand, prefer labile carbon compounds and organic nitrogen (Treseder et al. 2011), particularly in the form of plant root exudates, widely abundant in eutrophic, nutrient-rich soils (Balasooriya et al. 2014). In contrast to Gram-positive bacteria, resilience towards drought is less pronounced. In this sense, the intensification in both length and frequency of drought events can select drought-resistant microbes, changing soil microbial population fitness and composition [15, 16]. Thus, the observation that drought often induces a legacy effect, even after rewetting (Kaisermann et al. 2017), reducing soil bacterial richness in the long term is not surprising.

Taking into consideration ongoing climate change and its predicted impact on the global precipitation regime (Huntington 2006), investigating buffering factors of drought, is extremely relevant (Huang et al. 2023). One of those factors is plant community composition, which can influence and modulate the soil microbial community by recruiting and sustaining important microbial taxa (Hartman and Tringe 2019, Abedini et al. 2021). Previous work (Wang et al. 2019, Schmid et al. 2021) has already shown the beneficial effects of increasing plant richness on ecosystem functions (Isbell et al. 2015) and microbial community composition (Lange et al. 2015, Eisenhauer et al. 2017). Higher plant richness promotes nutrient turnover, biomass production, and overall ecosystem resilience against stress and disturbances (Roscher et al. 2004, Isbell et al. 2017). Biodiverse environments are also characterized by an increase in carbon and nitrogen stocks in soil, which ultimately contributes to higher productivity and ecosystem quality (Weisser et al. 2017, Yang et al. 2021). In this regard, both plant- and microbial communities are key to the maintenance of essential ecosystem functions, providing metabolic complementarity and stabilizing the overall ecosystem in response to drought (Vogel et al. 2012).

Increasing plant richness can increase microbial activity (Bartelt-Ryser et al. 2005, Lange et al. 2015) and possibly work as a mitigator of long-term drought effects on the soil microbiome. Regarding drought, plant community richness has already been shown to increase complementarity between plant species, with further adaptation to plant offspring after long periods of drought (Chen et al. 2022). A similar study, however, indicates that increasing plant diversity did not show any significant buffering effects on the soil fungal community, which significantly responded to long-term drought (Albracht et al. 2023). Although the effect of drought and shifts in precipitation regimes on plant communities has already been investigated (Zeppel et al. 2014), the complex interaction between plant diversity, microbial communities, and drought is poorly understood, especially in the phase of the recovery period after the drought event. Investigating the potential buffering effects of plant communities on soil microbial richness can be crucial to maintain critical ecosystem functions as affected by climate change.

To investigate whether plant richness changes recovery of bacterial communities one year after repeated summer droughts, we made use of an experimental gradient in plant species richness, which was established in 2002 in “The Jena Experiment” (Schmid et al. 2004, Weisser et al. 2017). Here we simulated recurrent summer droughts over 9 years by installing rain out shelters during every years summer season for 6 weeks (Vogel et al.2013a). Control plots were also sheltered to account for potential side effects of the roof infrastructure but received ambient amounts of precipitation. Bulk soil samples were taken from control and drought treatments one year after the last summer drought treatment from all 80 plots of the plant richness gradient. Metabarcoding was used to assess changes in bacterial and archaeal diversity and composition across the treatments and the plant species richness gradient. Our main hypotheses were that (i) drought reduces soil microbial richness and changes soil microbial community coposition in comparison to plots which received regular precipitation, even one year after the drought has been terminated and that (ii) increasing plant richness will buffer potential drought-induced soil microbial diversity loss.

## Materials and methods

### Experimental design and sampling

The drought experiment was established at the field site of the Jena Experiment (Schmid et al. 2004, Weisser et al. 2017), which is has been running since 2002. The site is located at the floodplain of the Saale River (50°55′43.61″N, 11°35′23.64″E, altitude 130 m a.s.l.) in Jena. The mean annual air temperature is 9.9°C (1980–2010), and mean annual precipitation is 610 mm (Hoffmann et al. 2014). The soil is classified as an Eutric Fluvisol (World Reference Base for Soil Resources 2015 (Weisser et al. 2017) with a pH value range from 7.1 to 8.4 and C_org_ 5–33 g C kg^−1^ (Roscher et al. 2004).

**Figure 1.**
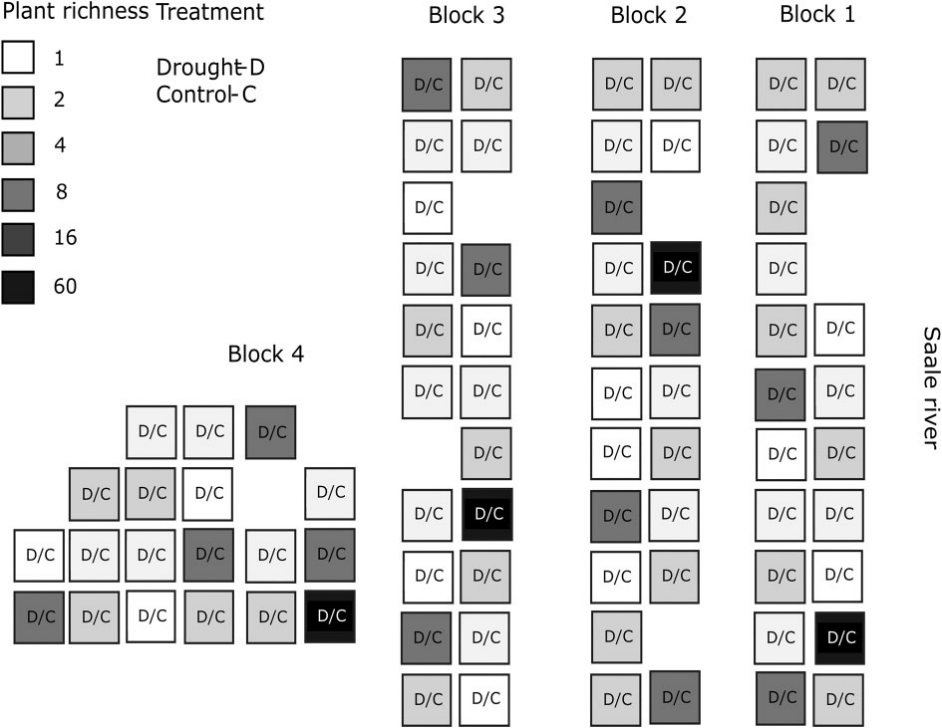
Schematic representation of the Jena Experiment field site with the split-plot design regarding the drought experiment. Drought exposed and control plots are nested inside the experimental plots with increasing plant richness. Spatial distribution resembles the plot distribution in the experiment field site.

The Jena Experiment consists of 80 plots (size 20 × 20 m). Those were planted with different plant community compositions in 2002. The plant communities vary in species richness (1, 2, 4, 8, 16, and 60 plant species) and number of plant functional groups (1–4 groups: grasses, small herbs, tall herbs, and legumes). Plant species richness in the manuscript is referring to this initially sown communities. Detailed information of composition and number of replicates per plant species richness level can be found in Tables S1. The plant species richness is maintained by two weeding campaigns a year and the plots are managed by two mowing events. The plots were randomly distributed among four blocks (Fig. 1) to account for spatial variations in edaphic properties (including soil texture and water-holding capacity), which are related to the distance of the plots to the adjacent river Saale. For example, sand content range from 6% to 48%, silt from 38% to 71% and clay from 13% to 25% (Fischer et al. 2015). In this framework the drought experiment was conducted from 2008 to 2016. Therefore, prior to the second annual mowing in mid-July, transparent rain shelters (wood and PVC sheets) were installed for 6 weeks every year on every plot (Vogel et al.2013a) covering two 1 × 1 m areas per plot. One roof covered the “drought” treatment, which received no water after installation (the ‘drought’ treatment), and one covered the “control” treatment, which received collected rain water as equivalent precipitation after rain events, thereby controlling for roofing effects such as altered light and temperature (Vogel et al. 2013b). The rain shelters reduced summer precipitation by an average of 42% in the period from 2008 to 2014 (Wagg et al. 2017).

As the only exception to the above described procedure, in 2013, the rain shelters were installed later (23^rd^ August to 23^rd^ September) and shorter (4, 5 weeks), because of a natural flooding event of the Saale River occurring from 30^th^ of May to 24^th^ of June (Cesarz et al. 2017).

To assess the changes in the soil microbial community after successive periods of drought, bulk soil from 0 to 15 cm was sampled in August 2017 (one year after the last experimental drought) from all plots and treatments (80 plots x 2 treatments). Therefore, 5 soil cores per treatment were pooled, resulting in approximately 50 g. The soil was sieved to 2 mm for homogenization and to remove bigger plant materials. Soil samples were stored at −80°C until processing for DNA extraction and metabarcoding.

### Soil moisture, pH and plant biomass

Soil moisture was measured gravimetrically. One gram of fresh soil was weighed (each sample was measured in duplicate) and left in the oven overnight at 104°C. After cooling, the soil was weighted several times until a stable weight was reached. The soil moisture is given in g of water/g of soil. pH was measured by adding 25 ml of 0.01 M calcium chloride to 10 g of air-dried soil, which was vigorously shaken. Samples were incubated at room temperature for 1 h, and then the pH was measured with a calibrated pH meter (Albracht et al. 2023). Total biomass was calculated by harvesting the plant biomass inside the 0.1 m^2^ subplot center of the 1 x 1 m plot. Samples were cut with scissors in the field at around 3 cm height above soil surface, stored in plastic bags at 4°C for transportation to the laboratory where the total dry biomass was weighted.

### DNA extraction and metabarcoding

DNA extraction from soil samples was performed using the DNeasy Powersoil Kit (Qiagen- Germany). The resulting DNA was quantified by a Qubit fluorometric system (Thermo—Germany), and the quality was checked using a Nanodrop photometric system (Thermo—Germany) and by gel electrophoresis. As a control for DNA extraction, we included a blank extraction (DNA extraction without sample).

For metabarcoding of bacterial communities we performed Illumina next-generation amplicon sequencing targeting the 16S rRNA gene using the primer pair 515F (Parada et al. 2016) and 806R (Apprill et al. 2015). Each reaction had 25 µL containing 12.5 µL NEB Next High-Fidelity Master Mix (Thermo—Germany), 0.5 µL of each primer at 10 pmol/µl, 2.5 µL of 3% BSA, 1 µl of 5 ng/µL diluted DNA, (for the negative control, 1 µl of DEPC-treated water instead) and 8 µL of DEPC-treated water. The amplification program was as follows: 98°C for 1 min, followed by 23 cycles of 98°C for 10 s, 55°C for 30 s and 72°C for 30 s, and a final extension at 72°C for 5 min. Samples were indexed using a Nextera® XT Index Kit v2 (Illumina—USA) and purified with MagSi-NGSprep Plus Beads (ratio 0.8 beads:1 sample) according to the manufacturer's protocol, and quality assessment was performed via a Fragment Analyzer (Agilent—Germany). High-quality DNA was diluted to 4 nM and sequenced on an Illumina MiSeq using a MiSeq Reagent v3 (600 Cycle) kit. PhiX (5 pM, 20%) was loaded alongside the samples. The raw sequencing reads were uploaded to NCBI sequencing read archive under the BioProject number PRJNA937585 and BioSample SAMN37746197.

### Bioinformatics

After sequencing, samples were uploaded to the European Galaxy server (https://usegalaxy.eu). The Cutadpat tool was used to remove adapters, and read quality was accessed via FastQC and with the dada2 version 1.16 (Callahan et al. 2016) plotQualityProfile option. Trimming parameters were set to 220 bp for forward reads and 200 bp for reverse reads, and dada2 was used to trim the sequences without adapters. We also used dada2 to apply error rates, merge the read pairs and make a sequence table according to the default dada2 pipeline (https://benjjneb.github.io/dada2/tutorial.html). Taxonomy was assigned using the “assingTaxonomy and addSpecies” function, aligning the Amplicon Sequencing Variants (ASVs) against the Silva database (Quast et al. 2013) version 138. The table with ASV counts and taxonomic assignments was downloaded, and all statistical analyses were conducted using the R environment (v4.2.2), implemented into the packages phyloseq v.1.4 (McMurdie and Holmes 2013), microbiome (v1.18) (Leo and Sudarshan 2017) and tidyverse (v1.3.1) (Wickham et al. 2019). First nonbacterial ASVs along with any ASV assigned to chloroplasts and mitochondria were removed. Further exogenous ASVs present in the negative controls using the prevalence-based method from package decontam (v1.16) (Davis et al. 2018) were excluded from further analysis and batch effects from multiple sequencing runs were addressed using the “ComBat_seq” function from package sva (v3.44) (Leek et al. 2012). Finally, nonsingleton ASVs observed in at least 5% of the samples were used for further analysis.

### Statistics and data visualization

We estimated microbial alpha diversity in each sample as richness by counting the number of ASVs and Inverse Simpson diversity index using the packages DivNet (v0.4) (Willis and Martin 2022) and breakaway (v4.7.9) (Willis and Bunge 2015). Differences in microbial beta diversity were estimated via PERMANOVA (999 permutations) over Bray-Curtis distance matrices (Oksanen et al. 2022). For that, we used the adonis2 function from package Vegan v2.6.2. Additionally, we calculated the effects of the constrains soil water content, pH, plant dry biomass on community composition by using standardized estimates of beta diversity to run Redundancy analysis (RDA) using MicroViz package (v 0.10.10) (Barnett et al. 2021). We visualized the distance across the samples by plotting the first and second components of a singular value decomposition of the count matrix.

We analyzed the differential abundance of ASVs between control and drought samples at each plant richness level using ANCOM-BC (v1.6) (Lin and Peddada 2020). We filtered significantly increased/decreased ASVs (*P* < 0.05), contrasting their abundances between control and drought-exposed plots across the plant richness gradient. The code and data used in these analyses are deposited in the GitHub repository https://github.com/rsiani/yuri_et_al_22.

For the statistical analyses, plant species richness and number of functional groups were log-transformed to improve distribution and reach linearity. We fit the transformed data to a linear mixed-effects model using the lme function in the package *nlme* to investigate the effects of block, plant species richness, number of functional groups, drought treatment and plot on the measured variables (Inverse Simpson and Observed Richness metrics for alpha diversity). The drought treatment was represented by subplots. The fitting order was model←lme(AlphaDiversityMetric∼Block+log(SpeciesRichness)*DroughtTreatment, random=∼1|Plot), moving the Block factor to a fixed term to investigate the changes in the outcome and changing Species Richness per Functional Groups to investigate the effects of functional groups instead of plant species richness. The linear mixed effect models analyzes also had the advantage to account for unbalanced data (in our case the 60 species richness level has less observations than the other groups—Tables S1) by incorporating both fixed and random effects, enabling flexible modeling of individual variability, handling unequal group sizes, and explicitly addressing the correlation structure within groups (Brown 2021).

## Results

### Drought and plant richness effects on soil bacterial communities

**Figure 2.**
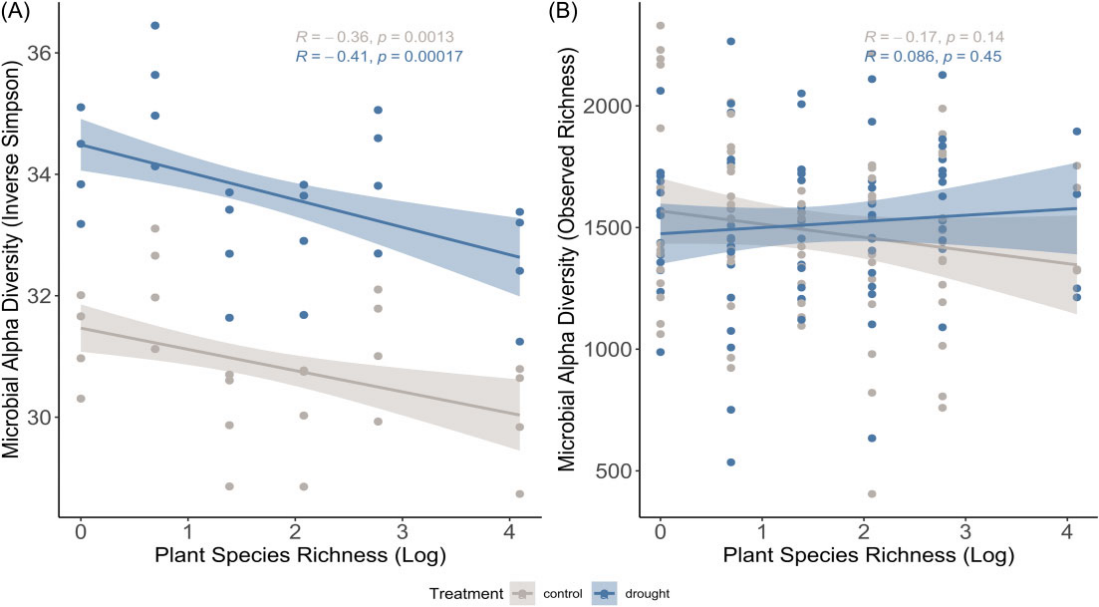
Pearson correlation plots between alpha diversity measurements (Inverse Simpson and Observed richness) against plant species richness (natural log). Drought plots are represented in blue color, while gray dots and lines represent the control plots. Drought plots present higher diversity for Inverse Simpson (A), while there is no clear differentiation between the treatments in the number of observed ASVs (B).

Linear mixed effects model fitting revealed a significant positive effect of the drought treatment and the plant richness gradient on the inverse Simpson index (*P* < 0.001). The number of observed ASVs (observed richness) was not significantly affected by the experimental design. The separation between drought and control for inverse Simpson can also be observed in the correlation plots (Fig. 2A), where drought-treated plots show higher dominance (blue dots) when compared to the control treatment (gray dots), while no clear separation could be observed in the number of observed ASVs (Fig. 2B). However, inverse Simpson negatively correlated with plant richness (*R* = −0.36 for control plots and *R* = −0.41 for drought plots–Fig. 2A).

To investigate the effects of the plant richness gradient on the drought effects, we performed a post hoc test, contrasting the means of control plots against drought plots using the Inverse Simpson and number of observed ASVs as response variables. First, we observed that the difference between control and drought treated plots is significant for Inverse Simpson along the plant richness gradient (*P* < 0.001–Table 1A), but not for richness (*P* = 0.7) in alignment with the trend observed in the correlation plots in Fig. 2. Secondly, the negative values of estimated marginal means while contrasting control against drought samples presents a consistent negative result, showing that average Inverse Simpson values are consistently higher in drought plots. We also observed that the ratio slightly decreases along the plant richness gradient, suggesting that the difference between drought and control plots decreased as the plant richness increased.

**Table 1.** (A) ANOVA table displaying the numerator and denominator degrees of freedom, alongside the F and p values for the linear mixed effects models (lme). We used Inverse Simpson and richness (number of observed ASVs) of soil bacterial communities as response variables. The fitting order was AlphaDiversity∼block+log(PlantRichness)*treatment, random=∼1|plot. (B) Estimated marginal means (EMMs) generated using the emmeans package in R. The statistical model employed was as previously described. Post hoc comparisons were conducted using Tukey's method, using pairwise comparison between control and drought across the plant diversity levels. We used the inverse Simpson and richness as microbial diversity metrics.

	Linear mixed effect models											
**A**	**Inverse Simpson**				**Richness**							
	**numDF**	**denDF**	**F-value**	***P*-value**	**numDF**	**denDF**	**F-value**	***P*-value**				
(Intercept)	1	76	128388.44	**<.0001**	1	76	2996.3293	**<.0001**				
Block	3	75	20.28	**<.0001**	3	75	0.7395	0.5680				
Plant Richness	1	75	23.07	**<.0001**	1	75	0.4644	0.4977				
Drought Treatment	1	76	28349.18	**<.0001**	1	76	0.1488	0.7008				
Plant richness and Drought	1	76	0.02	0.8895	1	76	2.5683	0.0904				
	**Post hoc analysis**											
**B**	**Inverse Simpson**						**Richness**					
**Contrast**	**Diversity lvl**	**estimate**	**SE**	**df**	**t.ratio**	***P*.value**	**Diversity lvl**	**estimate**	**SE**	**df**	**t.ratio**	***P*.value**
control—drought	1	−3.02	0.0257	76	−117.604	**<.0001**	1	93.8	92.3	76	1.016	1
control—drought	2	−2.97	0.0188	76	−157.432	**<.0001**	2	38.8	67.8	76	0.572	1
control—drought	4	−2.91	0.0153	76	−190.494	**<.0001**	4	−16.2	55.1	76	−0.294	1
control—drought	8	−2.86	0.0172	76	−166.478	**<.0001**	8	−71.2	61.8	76	−1.152	1
control—drought	16	−2.81	0.0232	76	−120.992	**<.0001**	16	−126.2	83.5	76	−1.512	0.6731
control—drought	60	−2.71	0.0389	76	−69.790	**<.0001**	60	−231.1	139.6	76	−1.655	0.6119

**Figure 3.**
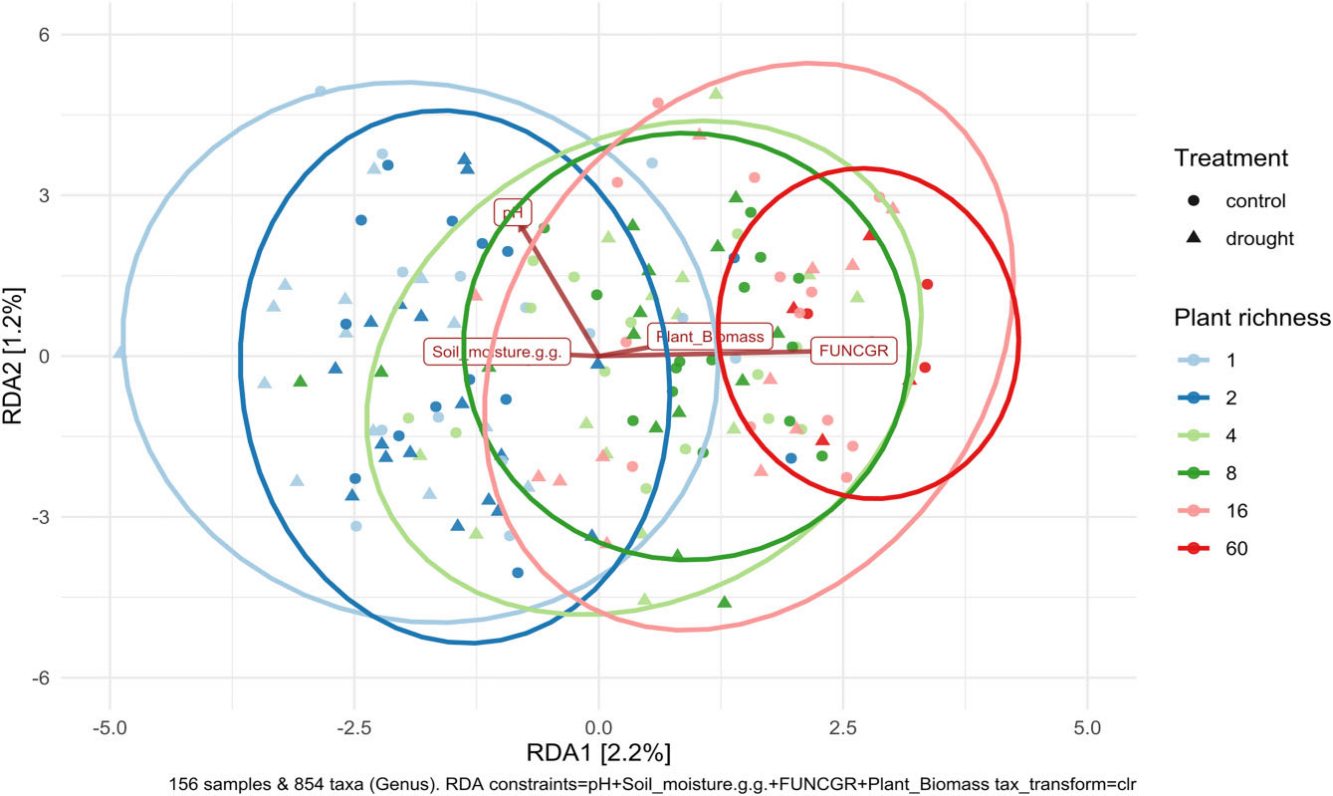
Redundancy analyses (RDA) plot displaying the relationships between pH, plant biomass, soil moisture, and number of plant functional groups as constrains. In the plot, the environmental variables (pH, plant biomass, soil moisture, and number of functional groups) are represented by arrows, indicating their direction and strength of influence. The length of the arrows represents the magnitude of the effect each variable has on the biological communities. The ellipses are colored according to the diversity level of each plot, and the dot shapes represent the drought treatment (circles for control and triangles for drought-treated plots).

To investigate the effects of the experimental design on soil bacterial composition, we used Redundancy Analysis (RDA) over Bray‒Curtis dissimilarity distance (Fig. 3) using soil moisture, soil pH, and plant dry biomass as explanatory variables. We observed that soil bacterial community composition changes along the plant richness gradient, and PERMANOVA results indicate a significant effect of both plant species richness and drought treatments on the bacterial community composition (*P* = 0.001 in both cases–Table 2). Although drought exerted a significant effect on soil bacterial composition, we can observe plant species richness to be the main driver of those bacterial communities (Fig. 3). This data also complements the results observed in the linear models and correlation plots for richness and dominance (Fig. 2 and Table 1), indicating that the impact of plant richness resides on the community composition, not on the overall diversity. Regarding the explanatory variables, we observed a positive correlation between the number of functional groups and plant richness, which is expected due to the increase in functional groups alongside the plant richness gradient in the Jena Experiment design. Soil moisture content displayed a slight negative correlation with the plant diversity gradient, while pH did not seem to be influenced by the same gradient (Fig. 3).

**Table 2.** PERMANOVAs over Bray‒Curtis dissimilarity distance displaying the effect size and the significance of each tested variable. Plant diversity and drought treatment both presented significant effects on bacterial community composition, while the interaction between both factors was not significant.

	Permanova (Bray‒Curtis distance)				
	Df	SumOfSqs	R^2^	F	Pr(>F)
Plant Species Richness	5	0.8097	0.05448	17.605	**0.001**
Drought Treatment	1	0.2312	0.01556	25.135	**0.005**
Plant Richness and Drought Treatment	5	0.3903	0.02626	0.8486	0.836
Residual	146	134.292	0.90370		
Total	157	148.603	100.000		

### Taxonomic responses to drought and plant species richness

We also investigated the effects of both plant species richness and summer drought on the taxonomical composition of soil bacterial communities. The taxonomical annotation of sequencing reads indicates that the overall bacterial community composition in our experiment was dominated by the same taxa, regardless of the drought treatment or plant richness level. Gemmatimonadota, Verrucomicrobiota, Patescibacteria, Myxococcota, Bacteroidota, Chloroflexi, Acidobacteriota, Actinobacteriota, Proteobacteria, and Planctomycetota were the most predominant phyla, being present in all treatments and all plant richness levels (Fig. 1).

**Figure 4.**
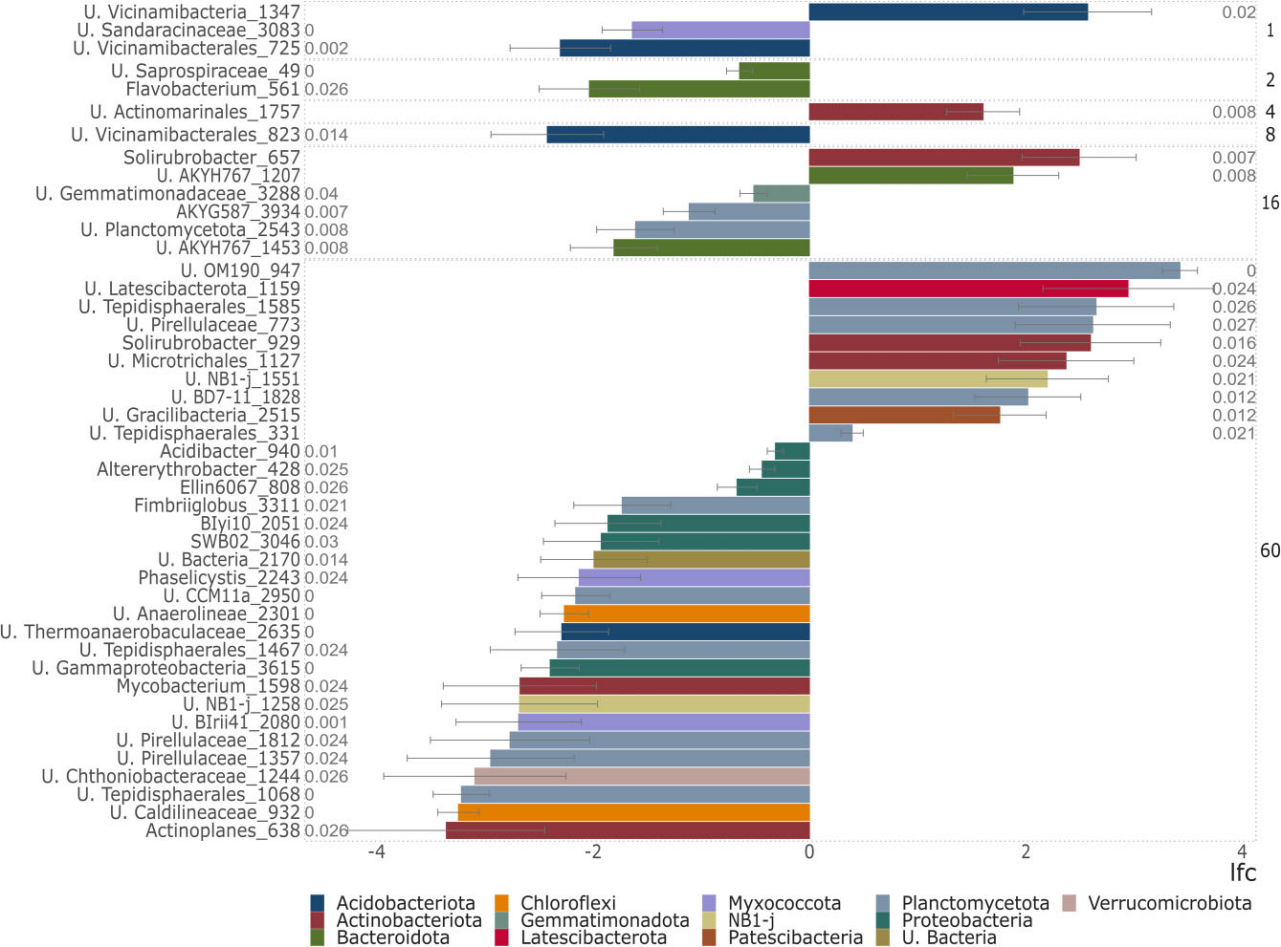
Differential analysis performed with ANCOMB package. The analysis estimated significant differences in ASV abundances between drought and control samples across the plant diversity gradient, with positively (right) and negatively (left) impacted taxa. Taxonomy on the left-hand side indicates the last possible level where that given ASV could be assigned, while bars are colored according to phylum (legend in the bottom). Plant diversity levels are indicated by the numbers on the right.

Differential abundance analysis (Fig. 4) reveled that monoculture plots had both increasing and decreasing ASVs belonging to the phylum Acidobacteriota with the drought treatment, both assigned to the Vicinamibacteria class, while 1 ASV assigned to the Myxococcota phylum was more abundant in the controls (Fig. 4). Plant richness levels 2 and 8 had both only significantly decreased ASVs after the drought treatment, assigned to Bacteroidota and Acidobacteriota phyla, respectively. On the other hand, 4-species richness level only presented a single ASV, which significantly increased under drought treatment. This ASV was assigned to the Actinoacteriota phylum. In the 16-species plots, two ASVs were significantly higher under drought and were assigned to Actinobacteriota and Bacteriodota. 4 ASVs declined under drought and belonged Gemmatimonadota, Plantomycetota and Bacteroidota. The 60-species richness level was the one with the most differential abundant ASVs with 33 ASVs. However, this might be an artifact caused by a lower number of replicated plots at this plant richness level (4 plots). The ASVs increased with the drought treatment were assigned to the Planctomycetota, Latescibacterota, Actinobacteriota and Patescibacteria phyla, while ASVs belonging to Proteobacteriota, Planctomycetota and Chloroflexi were severely reduced. Our analyzes revealed that the affected bacterial taxa were particular to each of the analyzed plant richness levels, without a consistent bacterial taxon being affected by the drought treatment consistently.

## Discussion

Our study investigated the effects of increasing plant richness on the recovery of soil bacterial communities after recurrent induced summer droughts. We demonstrated that drought consistently increased bacterial dominance in comparison to plots which received ambient precipitation, even one year after the drought was terminated. This increase was consistent along the plant richness gradient, which also significantly impacted the composition of soil bacterial communities.

### Bacterial diversity increases with long-term summer drought

The effects of drought events have already been largely studied in the soil sciences (Lipiec et al. 2013, Geng et al. 2015, Schimel 2018). Drought events have a drastic impact on plant and soil bacterial community abundance and composition. The lack of water increases temperature oscillations, reduces nutrient availability, and causes changes in the overall soil structure, making soils more compact and less porous (Lipiec et al. 2013, Zeppel et al. 2014, Geng et al. 2015, Chen et al. 2022). Those effects can impair plant growth and development and are largely detrimental for ecosystem functioning. Drought effects have also been largely demonstrated for soil bacterial communities (Schimel et al. 2007, Sardans and Pen 2008, Preece et al. 2019), with the microbial response to drought representing a major disturbing factor for soil ecosystem functioning. The lack of water impacts soil microbes in several ways, limiting nutrient availability (Lawrence et al. 2009, Carson et al. 2010), reducing community connectivity through dissolved molecules (Carson et al. 2010, Manzoni and Katul 2014, Manzoni et al. 2016), and, obviously, diminishing the availability of water as a resource itself, which is essential to the basic functioning and maintenance of cellular processes (Potts 2001). Taking this into consideration, a drop in soil bacterial diversity shortly after a drought event is expected. Besides that, drought events can quickly change soil properties and nutrient availability, a situation where copiotrophic bacterial taxa do not have enough time to react to drought exposure or simply do not possess the necessary machinery to cope with the changes imposed by drought.

In our study, however, we tested the recovery of soil bacterial community to long-term, repeated summer drought, and the effect of increasing plant species richness on this recovery. In contrast to acute drought, chronic exposure to drought stress imposes a different challenge to soil bacterial communities. The lack of water diminishes soil homogenization, isolating bacterial communities in smaller compartments, which promotes niche formation (Carson et al. 2010), species differentiation (Dumbrell et al. 2010), and an increase in bacterial metabolic dependency (Morris et al. 2012). The absence of water also leaves space for more air and therefore more oxygen (Preece et al. 2020), increasing the access of the soil bacterial communities to other gaseous and volatile substrates (Insam and Seewald 2010), such as methane. The extra oxygen and new substrate availability can then be used as high energy sources for soil bacteria to explore less available and more diverse substrates (Hartmann et al. 2011, Fest et al. 2017).

Our data indicate that exposure to long-term, repeated drought has a lingering effect on soil bacterial community composition. Soil samples taken 1 year after the termination of drought exposure show that diversity of soil bacterial communities increases with the drought treatment. This trend has already been observed in soils from natural holm oak forest exposed to chronic drought (Preece et al. 2019); however, it has—to our knowledge—never been reported in grasslands. We observed significant effects of drought treatment on the inverse Simpson (dominance) diversity index (*P* < 0.001 in both cases), while richness (the number of observed ASVs) did not show any significant responses to the drought treatment (*P* = 0.7). This indicates that the number of taxa did not differ between the treatments; but that the importance of dominant taxa increases in the drought treatment. This trend indicates long-lasting shifts in dominant species in each treatment, as observed in the taxonomic composition analysis. Drought is also shown to significantly affect the composition of soil bacterial community, as observed on the PERMANOVA calculations (*P* = 0.005).

A possible explanation for the higher inverse Simpson index under drought might be the fragmentation of bacterial communities in the soil as a result of niche separation, alongside the promotion of less competitive bacteria in this less connected environment. Treves et al. (2003) demonstrated that less dominant taxa have a better chance of establishing as soil moisture decreases (Treves et al. 2003), with the competition between highly abundant taxa and less abundant taxa being more even under this condition. In this scenario, drought can reduce the nutrient availability to fast-growing taxa, allowing the growth of fastidious, less abundant taxa. Similarly, Carson et al. (2010) demonstrated that bacterial diversity increased in soils with low water content in comparison with the same soils with higher moisture (Carson et al. 2010). The changes in this case could be attributed to low pore connectivity due to the lack of water, increasing spatial isolation and reducing soil homogenization. In this sense, our experiment indicates a persistent difference existing in bacterial communities after 9 years of repeated drought, even 1 year after the last treatment period, which indicates a persistent, long-term legacy effect of moderate droughts on soil bacterial communities. Since plots have only been covered from precipitation during summer months, the breaks in between (other seasons when both drought exposed and control plots received equal amounts of precipitation) did not represented a sufficiently long recovering time, since drought effects could still be observed. In line with our data, Vogel et al. (2013a) observed a significant effect of drought treatment on the litter decomposition, also irrespective of plant richness. Comparing to our context, we observed a highly significant effect of drought treatment on the soil bacterial dominance, with small correlation with the plant richness gradient.

However, other publications on the same experiment described contrasting effects, in comparison with our data. Albracht et al. (2023) working on the same experiment, investigated the effects of both plant richness increase and drought treatment on the diversity and composition of arbuscular mycorrhiza (AMF) and total fungal community. They reported a significant impact of the plant richness gradient on the diversity and composition of AMF and total fungal community but did not observe any significant effect of drought treatment on the same variables. Wagg et al. (2017) observed a reduction of plant biomass on drought treated plots in comparison to control plots, however reported a less pronounced biomass loss as the plant richness increases. Interestingly, biomass measurements taken in the same year of the soil sampling for this experiment (2017) did not show any significant effect of drought treatment over total plant biomass (Fig. S2 and Table S3) anymore, indicating that recovery of bacterial communities to the drought stress does not necessarily follow the patterns observed on the plant communities. Regardless of this data, we cannot completely exclude an interaction between changes in the recruitment of soil endophytic bacteria and the reduction of selective pressure caused by the changes in plant-soil feedbacks, especially since our data does not discriminate species-specific biomass or functional groups.

### Plant richness gradient changes soil bacterial composition

Biodiversity is crucial for ecosystem resilience, which refers to the ability of an ecosystem to withstand and recover from disturbances (Cardinale et al. 2012). A diverse range of species provides functional redundancy, ensuring that ecosystem processes and services are maintained even if some species are lost (Yachi and Loreau 1999). Biodiversity also enhances ecosystem resistance by reducing competition through niche complementarity and increasing adaptability through a broader genetic pool. In this sense, plant and soil bacterial communities are intimately linked, with plant communities directly impacting and modulating soil bacterial communities (Liu et al. 2020). This modulation takes place through diversification of plant exudates in soil (Eisenhauer et al. 2017, El Moujahid et al. 2017), which can be used as a substrate for bacterial growth, as well as the recruitment of specific bacterial taxa to complement plant growth needs, such as phosphorus and nitrogen supply (Berihu et al. 2023). In drought-exposed soils, increasing plant diversity reportedly improves ecosystem resistance and resilience (Wagg et al. 2017), mitigating the effects of drought on biomass loss with compensatory growth after rewetting (Wagg et al. 2017). However, the interaction between drought, the soil bacterial community and plant diversity is still poorly explored.

The effect of increasing plant richness varied according to the diversity metric analyzed. We could not observe a direct effect of plant richness on bacterial richness. We did, however, observe significant effects of plant richness gradient on the Inverse Simpson metric, as well as slightly negative correlation between this diversity metric and the plant richness gradient, which indicates that diversity calculated by Inverse Simpson slightly decreases along the plant richness gradient. This might be explained by the fact that Inverse Simpson places more emphasis on the dominance or concentration of individuals (He and Biswas 2019). Therefore, we estimate that the decrease in inverse Simpson indicated a higher proportion of dominant taxa in monocultures. The impact of plant richness gradient on the soil samples can also be observed on the PERMANOVA analysis, which shows significant effects of both plant richness gradient and drought treatment Together with the negative correlation observed between plant richness gradient, this data can indicated that increasing plant richness can favor the dominance of specific microbial species. Moreover, previous works on the Jena experiment observed that biodiversity effects on belowground environments might not be significant even though aboveground effects can be observed (Bessler et al. 2009), which can indicate that diversity effects on soil bacterial diversity can be confounded by other environmental factors.

The identity of the most abundant phyla did not change across the plant richness levels or between drought and control plots (Fig. S1). Soil samples were dominated by Actinobacteria, Acidobacteria, and Proteobacteria phyla, groups commonly abundant in grassland soil samples (Janssen 2006, Fierer et al. 2007). The ANCOMB analyses (Fig. 4), however, identified ASVs which significantly increased/decreased after the drought treatment along the plant richness gradient. The selection and increase/decrease in ASV abundance does not seem to be exclusive to a single phylum, since we could observe different genera inside of the same phylum being depleted, while others are increased. The Actinobacteriota phylum, for example, had phyla with a 2-fold increase in plant richness levels 4 and 16 (Actinomarinales order and *Solirubrobacter* genus, respectively), while in richness level 60 two of the present ASVs were depleted (*Mycobacterium* and *Actinoplanes* genera) while two others were increased (*Solirubrobacter* genus and Microtrichales order). Pérez Castro et al. (2019) also observed the decrease in Proteobacteria, Verrucomicrobia, and Acidobacteria, while Actinobacteria abundance increased after drought stress. Therefore, despite the observation of a significant effect on the overall bacterial diversity, the drought treatment in our experiment did not select any specific group or taxa.

This preferential accumulation of specific taxa according to plant diversity level indicates that the complementarity between bacterial and plant metabolism in the face of drought follows individual interactions at the level of bacterial species or even strains l, even though the invers Simpson responses to drought seem to be consistent despite the plant richness levels. A possible explanation for these patterns might be the variation in the number of plant functional groups implemented in parallel in the Jena Experiment (see Table S1) and/or the variation on species on each richness level, being the changes on soil bacterial composition a specie-specific interaction, instead of an overall response to the increase in surrounding diversity. The drought experiment conducted by Preece et al. (2019) also observed high variability in fungal community composition as affected by long-term drought, representing the most affected taxa highly dependent according to their experiment design. As previously mentioned, Albracht et al. (2023) observed the opposite: non- significant effect of drought and significant effect of plant richness gradient over fungal community. Together, this data indicates that microbial response to drought is dynamic, changes according to the investigated microbial group and according to the experimental design.

### Conclusion and outlook

In summary, our data indicates that the soil bacterial community diversity was increased after long-term drought, with a rather stable response to the plant richness gradient. This response might be explained by the spatial isolation of soil bacterial communities promoted by a reduction in water potential in comparison to control samples. Changes in community composition along the plant richness gradient were observed as changes in community profile (beta diversity) instead of overall community diversity (alpha diversity) indicating that individual plant-microbe interactions might prevail over community richness as determining factor for soil microbial community modulation. Those findings indicate that soil bacterial diversity can adapt to long term drought conditions, being affected by the increase in plant richness, which might have important consequences for ecosystem functioning in a changing climate. In this regard, future works might address the mechanisms behind those patterns, approaching the topic with different complementary methodologies (like transcriptomic and/or metabolomic studies) and approaching different aspects of the drought stress in soil microbial communities, as the long term recovery from stress, for example.

## Supplementary Material

fiae096_Supplemental_File
