# Supplementary material for: The effect of successive summer drought periods on bacterial diversity along a plant species richness gradient
Source: FEMS Microbiol Ecol. 2024 Jul 2;100(8):fiae096. doi: 10.1093/femsec/fiae096 (PMC11264299; doi:10.1093/femsec/fiae096)
Supplement: fiae096_Supplemental_File [file fiae096_supplemental_file.docx]

# **Supplementary Material**

The effect of plant species richness on the recovery of bacterial diversity after successive summer drought periods

Yuri Pinheiro ^1,2^; Roberto Siani ^1,2^; Cynthia Albracht ^3,4^, Yuanyuan Huang ^5,6^, Nico Eisenhauer ^5,6^, Anja Vogel ^5,6^, Cameron Wagg ^7,8^; Michael Schloter ^1,2^; Stefanie Schulz ^1^

^1^ Helmholtz Zentrum München, Research Unit Comparative Microbiome Analysis, Neuherberg, Germany

^2^ Technische Universität München, TUM School of Life Science, Chair of Environmental Microbiology, Neuherberg, Germany

^3^Swammerdam Institute of Life Sciences at University of Amsterdam, Amsterdam, the Netherlands

^4^Department Soil Ecology, Helmholtz Centre for Environmental Research – UFZ, Halle (Saale),

Germany

^5^ German Centre for Integrative Biodiversity Research (iDiv) Halle-Jena-Leipzig, Leipzig, Germany

^6^ Institute of Biology, Leipzig University, Leipzig, Germany

^7^ Department of Geography, Remote Sensing Laboratories, University of Zürich, Winterthurerstrasse

190, CH-8057, Zürich, Switzerland

^8^ Fredericton Research and Development Centre, Agriculture and Agri-Food Canada, 95 Innovation

Road, Post Office Box 20280, Fredericton, E3B 4Z7, NB, Canada

Supplementary Table S1: Detailed information about the experiment design. The desing consists of an split plot desing where drought treated samples and control samples were both nested inside the same plot. Each plot varies in plant species richness (monoculture, 2, 4, 8, 16 and 60 species) and the composition of functional groups (1, 2, 3 and 4 groups divide among grasses, small herbs, tall herbs and legumes).

| **Plant Species Richness** | **N of functional groups** | **Block** | **Plot** | **Treatment** | **Grasses** | **Small Herbs** | **Tall Herbs** | **Legumes** |
| --- | --- | --- | --- | --- | --- | --- | --- | --- |
| 1 | 1 | B1 | 8 | Control | 0 | 0 | 0 | 1 |
| 1 | 1 | B1 | 8 | Drought | 0 | 0 | 0 | 1 |
| 1 | 1 | B1 | 15 | Control | 0 | 0 | 1 | 0 |
| 1 | 1 | B1 | 15 | Drought | 0 | 0 | 1 | 0 |
| 1 | 1 | B1 | 18 | Control | 0 | 1 | 0 | 0 |
| 1 | 1 | B1 | 18 | Drought | 0 | 1 | 0 | 0 |
| 1 | 1 | B2 | 4 | Control | 0 | 0 | 1 | 0 |
| 1 | 1 | B2 | 4 | Drought | 0 | 0 | 1 | 0 |
| 1 | 1 | B2 | 5 | Control | 1 | 0 | 0 | 0 |
| 1 | 1 | B2 | 5 | Drought | 1 | 0 | 0 | 0 |
| 1 | 1 | B2 | 13 | Control | 0 | 1 | 0 | 0 |
| 1 | 1 | B2 | 13 | Drought | 0 | 1 | 0 | 0 |
| 1 | 1 | B2 | 15 | Control | 0 | 0 | 0 | 1 |
| 1 | 1 | B2 | 15 | Drought | 0 | 0 | 0 | 1 |
| 1 | 1 | B3 | 1 | Control | 0 | 0 | 1 | 0 |
| 1 | 1 | B3 | 1 | Drought | 0 | 0 | 1 | 0 |
| 1 | 1 | B3 | 6 | Control | 1 | 0 | 0 | 0 |
| 1 | 1 | B3 | 6 | Drought | 1 | 0 | 0 | 0 |
| 1 | 1 | B3 | 12 | Control | 0 | 0 | 0 | 1 |
| 1 | 1 | B3 | 12 | Drought | 0 | 0 | 0 | 1 |
| 1 | 1 | B3 | 17 | Control | 0 | 1 | 0 | 0 |
| 1 | 1 | B3 | 17 | Drought | 0 | 1 | 0 | 0 |
| 1 | 1 | B4 | 9 | Control | 0 | 0 | 0 | 1 |
| 1 | 1 | B4 | 9 | Drought | 0 | 0 | 0 | 1 |
| 1 | 1 | B4 | 12 | Control | 1 | 0 | 0 | 0 |
| 1 | 1 | B4 | 12 | Drought | 1 | 0 | 0 | 0 |
| 1 | 1 | B4 | 13 | Control | 0 | 0 | 1 | 0 |
| 1 | 1 | B4 | 13 | Drought | 0 | 0 | 1 | 0 |
| 2 | 1 | B1 | 5 | Control | 0 | 0 | 0 | 2 |
| 2 | 1 | B1 | 5 | Drought | 0 | 0 | 0 | 2 |
| 2 | 1 | B1 | 7 | Control | 0 | 0 | 2 | 0 |
| 2 | 1 | B1 | 7 | Drought | 0 | 0 | 2 | 0 |
| 2 | 2 | B1 | 16 | Control | 1 | 1 | 0 | 0 |
| 2 | 2 | B1 | 16 | Drought | 1 | 1 | 0 | 0 |
| 2 | 2 | B1 | 17 | Control | 1 | 0 | 1 | 0 |
| 2 | 2 | B1 | 17 | Drought | 1 | 0 | 1 | 0 |
| 2 | 3 | B1 | 19 | Control | 2 | 1 | 1 | 0 |
| 2 | 3 | B1 | 19 | Drought | 2 | 1 | 1 | 0 |
| 2 | 1 | B2 | 2 | Control | 2 | 0 | 0 | 0 |
| 2 | 1 | B2 | 2 | Drought | 2 | 0 | 0 | 0 |
| 2 | 2 | B2 | 8 | Control | 0 | 0 | 1 | 1 |
| 2 | 2 | B2 | 8 | Drought | 0 | 0 | 1 | 1 |
| 2 | 1 | B2 | 19 | Control | 0 | 2 | 0 | 0 |
| 2 | 1 | B2 | 19 | Drought | 0 | 2 | 0 | 0 |
| 2 | 2 | B2 | 20 | Control | 0 | 1 | 0 | 1 |
| 2 | 2 | B2 | 20 | Drought | 0 | 1 | 0 | 1 |
| 2 | 2 | B3 | 2 | Control | 1 | 0 | 1 | 0 |
| 2 | 2 | B3 | 2 | Drought | 1 | 0 | 1 | 0 |
| 2 | 1 | B3 | 8 | Control | 2 | 0 | 0 | 0 |
| 2 | 1 | B3 | 8 | Drought | 2 | 0 | 0 | 0 |
| 2 | 2 | B3 | 19 | Control | 1 | 1 | 0 | 0 |
| 2 | 2 | B3 | 19 | Drought | 1 | 1 | 0 | 0 |
| 2 | 1 | B3 | 21 | Control | 0 | 0 | 0 | 2 |
| 2 | 1 | B3 | 21 | Drought | 0 | 0 | 0 | 2 |
| 2 | 1 | B4 | 14 | Control | 0 | 2 | 0 | 0 |
| 2 | 1 | B4 | 14 | Drought | 0 | 2 | 0 | 0 |
| 2 | 2 | B4 | 15 | Control | 0 | 1 | 0 | 1 |
| 2 | 2 | B4 | 15 | Drought | 0 | 1 | 0 | 1 |
| 2 | 1 | B4 | 17 | Control | 0 | 0 | 2 | 0 |
| 2 | 1 | B4 | 17 | Drought | 0 | 0 | 2 | 0 |
| 2 | 2 | B4 | 21 | Control | 0 | 0 | 1 | 1 |
| 2 | 2 | B4 | 21 | Drought | 0 | 0 | 1 | 1 |
| 4 | 4 | B1 | 4 | Control | 1 | 1 | 1 | 1 |
| 4 | 4 | B1 | 4 | Drought | 1 | 1 | 1 | 1 |
| 4 | 1 | B1 | 13 | Control | 0 | 0 | 0 | 4 |
| 4 | 1 | B1 | 13 | Drought | 0 | 0 | 0 | 4 |
| 4 | 2 | B1 | 21 | Control | 2 | 0 | 2 | 0 |
| 4 | 2 | B1 | 21 | Drought | 2 | 0 | 2 | 0 |
| 4 | 4 | B2 | 1 | Control | 1 | 1 | 1 | 1 |
| 4 | 4 | B2 | 1 | Drought | 1 | 1 | 1 | 1 |
| 4 | 2 | B2 | 6 | Control | 0 | 2 | 0 | 2 |
| 4 | 2 | B2 | 6 | Drought | 0 | 2 | 0 | 2 |
| 4 | 1 | B2 | 9 | Control | 0 | 4 | 0 | 0 |
| 4 | 1 | B2 | 9 | Drought | 0 | 4 | 0 | 0 |
| 4 | 3 | B2 | 16 | Control | 0 | 2 | 1 | 1 |
| 4 | 3 | B2 | 16 | Drought | 0 | 2 | 1 | 1 |
| 4 | 3 | B3 | 3 | Control | 1 | 1 | 0 | 2 |
| 4 | 3 | B3 | 3 | Drought | 1 | 1 | 0 | 2 |
| 4 | 2 | B3 | 11 | Control | 2 | 2 | 0 | 0 |
| 4 | 2 | B3 | 11 | Drought | 2 | 2 | 0 | 0 |
| 4 | 1 | B3 | 13 | Control | 4 | 0 | 0 | 0 |
| 4 | 1 | B3 | 13 | Drought | 4 | 0 | 0 | 0 |
| 4 | 4 | B3 | 23 | Control | 1 | 1 | 1 | 1 |
| 4 | 4 | B3 | 23 | Drought | 1 | 1 | 1 | 1 |
| 4 | 4 | B4 | 4 | Control | 1 | 1 | 1 | 1 |
| 4 | 4 | B4 | 4 | Drought | 1 | 1 | 1 | 1 |
| 4 | 2 | B4 | 7 | Control | 0 | 0 | 2 | 2 |
| 4 | 2 | B4 | 7 | Drought | 0 | 0 | 2 | 2 |
| 4 | 3 | B4 | 11 | Control | 1 | 0 | 2 | 1 |
| 4 | 3 | B4 | 11 | Drought | 1 | 0 | 2 | 1 |
| 4 | 1 | B4 | 22 | Control | 0 | 0 | 4 | 0 |
| 4 | 1 | B4 | 22 | Drought | 0 | 0 | 4 | 0 |
| 8 | 2 | B1 | 2 | Control | 4 | 0 | 4 | 0 |
| 8 | 2 | B1 | 2 | Drought | 4 | 0 | 4 | 0 |
| 8 | 3 | B1 | 3 | Drought | 3 | 3 | 0 | 2 |
| 8 | 1 | B1 | 12 | Control | 0 | 0 | 0 | 8 |
| 8 | 1 | B1 | 12 | Drought | 0 | 0 | 0 | 8 |
| 8 | 4 | B1 | 14 | Control | 2 | 2 | 2 | 2 |
| 8 | 4 | B1 | 14 | Drought | 2 | 2 | 2 | 2 |
| 8 | 1 | B2 | 12 | Control | 0 | 0 | 8 | 0 |
| 8 | 1 | B2 | 12 | Drought | 0 | 0 | 8 | 0 |
| 8 | 4 | B2 | 14 | Control | 2 | 2 | 2 | 2 |
| 8 | 4 | B2 | 14 | Drought | 2 | 2 | 2 | 2 |
| 8 | 2 | B2 | 17 | Control | 0 | 4 | 0 | 4 |
| 8 | 2 | B2 | 17 | Drought | 0 | 4 | 0 | 4 |
| 8 | 3 | B2 | 21 | Control | 0 | 2 | 3 | 3 |
| 8 | 3 | B2 | 21 | Drought | 0 | 2 | 3 | 3 |
| 8 | 1 | B3 | 4 | Control | 8 | 0 | 0 | 0 |
| 8 | 1 | B3 | 4 | Drought | 8 | 0 | 0 | 0 |
| 8 | 3 | B3 | 5 | Control | 3 | 0 | 2 | 3 |
| 8 | 3 | B3 | 5 | Drought | 3 | 0 | 2 | 3 |
| 8 | 4 | B3 | 7 | Control | 2 | 2 | 2 | 2 |
| 8 | 4 | B3 | 7 | Drought | 2 | 2 | 2 | 2 |
| 8 | 2 | B3 | 20 | Control | 0 | 0 | 4 | 4 |
| 8 | 2 | B3 | 20 | Drought | 0 | 0 | 4 | 4 |
| 8 | 1 | B4 | 6 | Control | 0 | 8 | 0 | 0 |
| 8 | 1 | B4 | 6 | Drought | 0 | 8 | 0 | 0 |
| 8 | 2 | B4 | 8 | Control | 4 | 4 | 0 | 0 |
| 8 | 2 | B4 | 8 | Drought | 4 | 4 | 0 | 0 |
| 8 | 3 | B4 | 10 | Control | 2 | 3 | 3 | 0 |
| 8 | 3 | B4 | 10 | Drought | 2 | 3 | 3 | 0 |
| 8 | 4 | B4 | 16 | Control | 2 | 2 | 2 | 2 |
| 8 | 4 | B4 | 16 | Drought | 2 | 2 | 2 | 2 |
| 16 | 4 | B1 | 1 | Control | 4 | 4 | 4 | 4 |
| 16 | 4 | B1 | 1 | Drought | 4 | 4 | 4 | 4 |
| 16 | 2 | B1 | 6 | Control | 8 | 0 | 8 | 0 |
| 16 | 2 | B1 | 6 | Drought | 8 | 0 | 8 | 0 |
| 16 | 1 | B1 | 11 | Control | 0 | 0 | 16 | 0 |
| 16 | 1 | B1 | 11 | Drought | 0 | 0 | 16 | 0 |
| 16 | 3 | B1 | 20 | Control | 0 | 6 | 5 | 5 |
| 16 | 3 | B1 | 20 | Drought | 0 | 6 | 5 | 5 |
| 16 | 2 | B2 | 10 | Control | 8 | 8 | 0 | 0 |
| 16 | 2 | B2 | 10 | Drought | 8 | 8 | 0 | 0 |
| 16 | 4 | B2 | 18 | Control | 4 | 4 | 4 | 4 |
| 16 | 4 | B2 | 18 | Drought | 4 | 4 | 4 | 4 |
| 16 | 3 | B2 | 22 | Control | 5 | 0 | 5 | 6 |
| 16 | 3 | B2 | 22 | Drought | 5 | 0 | 5 | 6 |
| 16 | 1 | B3 | 9 | Control | 16 | 0 | 0 | 0 |
| 16 | 1 | B3 | 9 | Drought | 16 | 0 | 0 | 0 |
| 16 | 2 | B3 | 16 | Control | 0 | 8 | 0 | 8 |
| 16 | 2 | B3 | 16 | Drought | 0 | 8 | 0 | 8 |
| 16 | 4 | B3 | 22 | Control | 4 | 4 | 4 | 4 |
| 16 | 4 | B3 | 22 | Drought | 4 | 4 | 4 | 4 |
| 16 | 3 | B3 | 24 | Control | 6 | 5 | 0 | 5 |
| 16 | 3 | B4 | 2 | Control | 5 | 5 | 6 | 0 |
| 16 | 3 | B4 | 2 | Drought | 5 | 5 | 6 | 0 |
| 16 | 4 | B4 | 18 | Control | 4 | 4 | 4 | 4 |
| 16 | 4 | B4 | 18 | Drought | 4 | 4 | 4 | 4 |
| 16 | 2 | B4 | 20 | Control | 0 | 0 | 8 | 8 |
| 16 | 2 | B4 | 20 | Drought | 0 | 0 | 8 | 8 |
| 60 | 4 | B1 | 22 | Control | 16 | 12 | 20 | 12 |
| 60 | 4 | B1 | 22 | Drought | 16 | 12 | 20 | 12 |
| 60 | 4 | B2 | 3 | Control | 16 | 12 | 20 | 12 |
| 60 | 4 | B2 | 3 | Drought | 16 | 12 | 20 | 12 |
| 60 | 4 | B3 | 14 | Control | 16 | 12 | 20 | 12 |
| 60 | 4 | B3 | 14 | Drought | 16 | 12 | 20 | 12 |
| 60 | 4 | B4 | 1 | Control | 16 | 12 | 20 | 12 |
| 60 | 4 | B4 | 1 | Drought | 16 | 12 | 20 | 12 |


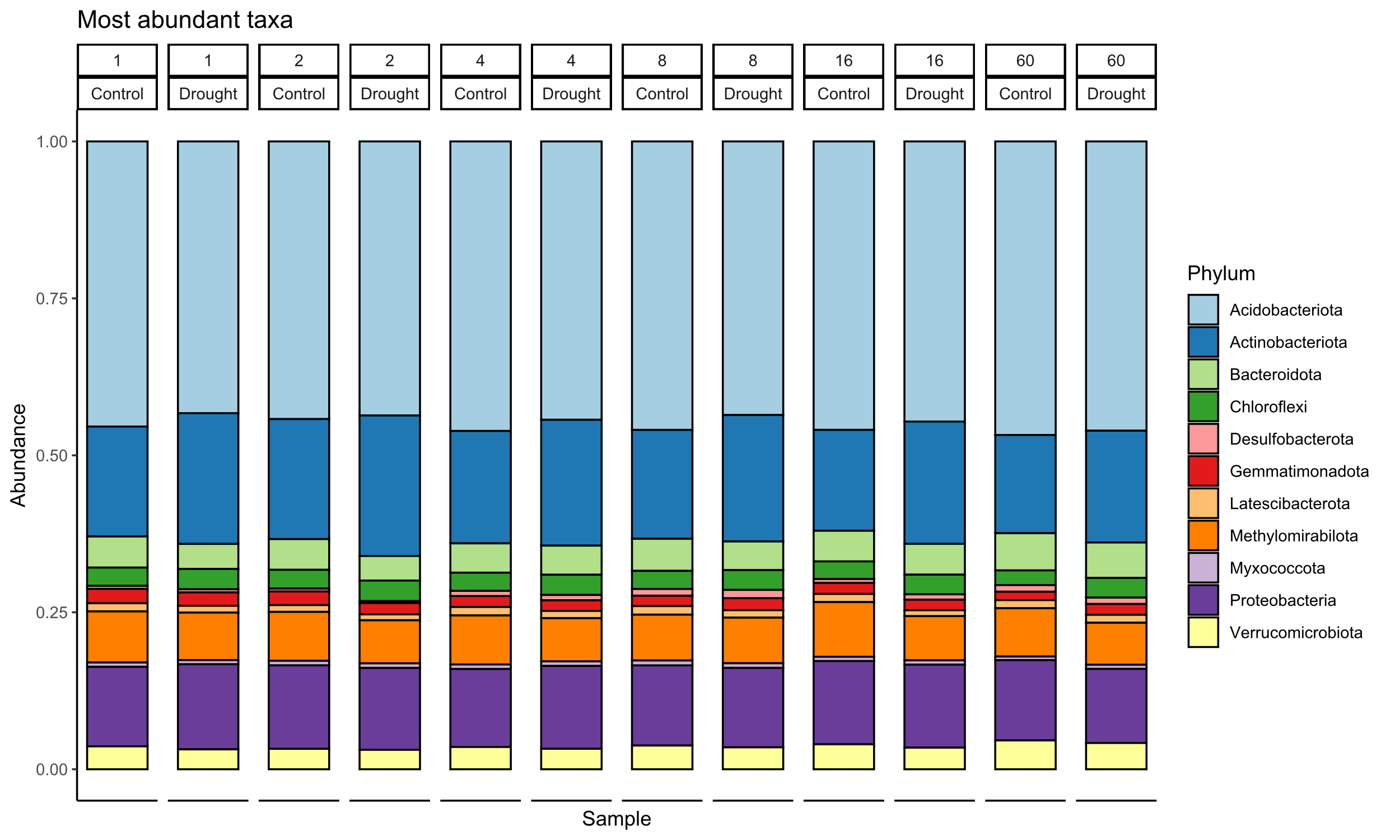


Supplementary Figure S1: Bar plot displaying the relative abundance of top 12 taxa at phylum level. Samples were merged at richness and treatment level. Overall bacterial diversity is stable along the richness levels and treatment, being dominated by Acidobacteria (especially Vicinamibacteraceae family) Actinobacteria, Proteobacteria and Bacteroidota. The taxa with significantly differs between the control and drought plots are displayed on the main text, figure 3.


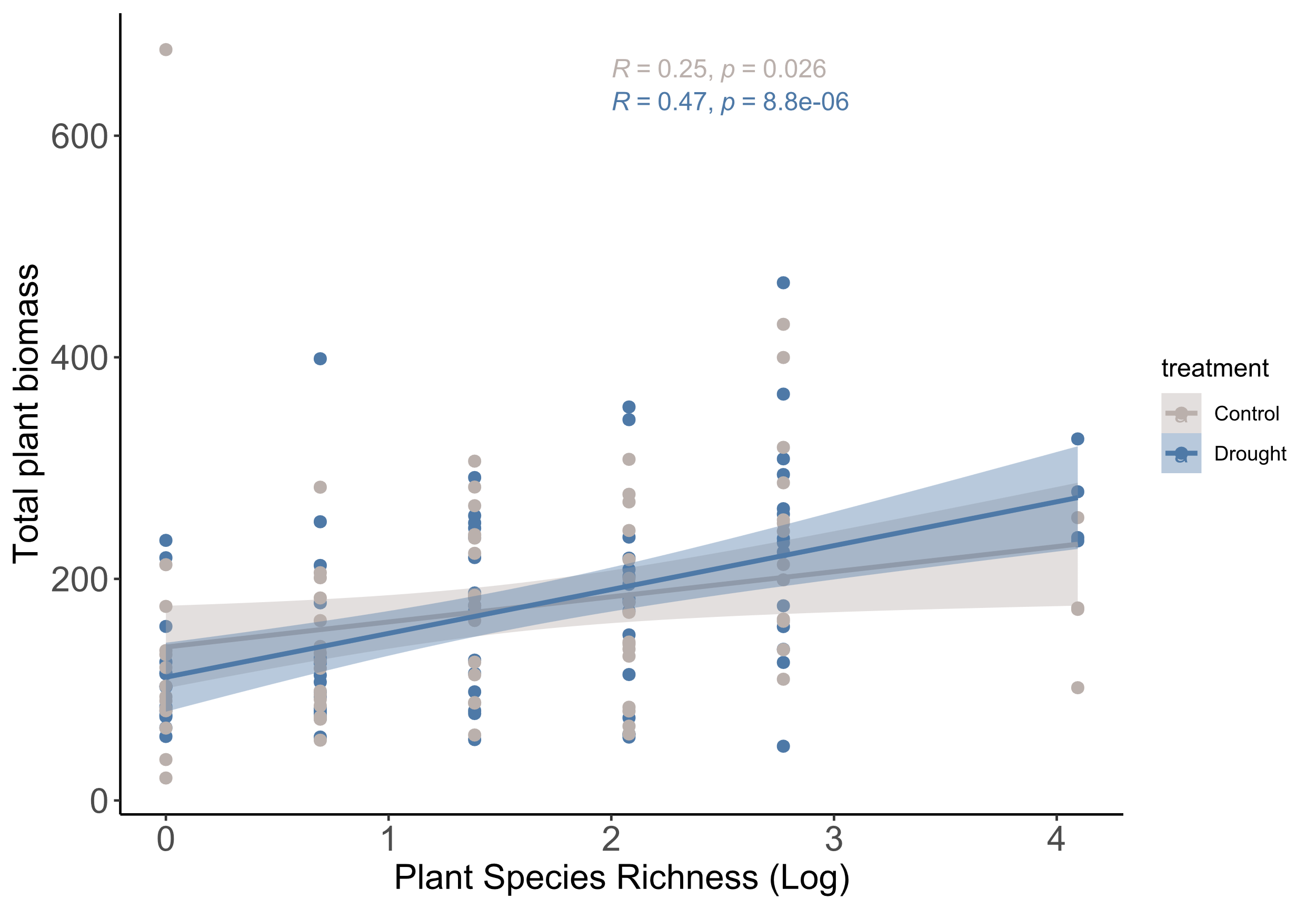


Supplementary Figure S2: Correlation plot displaying the relationship between plant scpecies richness and total plant biomass on the time of sampling from soil samples (August 2017). We observed a slightly positive correlation between the plant species richness gradient and the plant biomass production, however drought treatment did not impact biomass estimation after 1 year of termination of the drought treatment.

Supplementary table S3: table displaying the result of linear mixed effect models analyzes over total biomass data. We observed highy significant effect of plant richness on the over total biomass productivity while the drought treatment did not show significant effects. The fitting order of the terms in the model was TotalBiomass~Block+DroughtTreatment+log(PlantRichness)

|  | numDF | denDF | F-value | p-value |
| --- | --- | --- | --- | --- |
| Intercept | 1 | 131 | 573.2667 | <.0001 |
| block | 3 | 131 | 0.3629 | 0.7799 |
| treatment | 1 | 131 | 0.0000 | 1.000 |
| Plant richness | 1 | 131 | 263.8730 | <.0001 |
